# Short hairpin RNA-mediated knockdown of protein expression in *Entamoeba histolytica*

**DOI:** 10.1186/1471-2180-9-38

**Published:** 2009-02-17

**Authors:** Alicia S Linford, Heriberto Moreno, Katelyn R Good, Hanbang Zhang, Upinder Singh, William A Petri

**Affiliations:** 1 Department of Microbiology, University of Virginia, Charlottesville, Virginia, USA; 2 Department of Medicine, University of Virginia, Charlottesville, Virginia, USA; 3 Department of Internal Medicine, Division of Infectious Diseases, Stanford University School of Medicine, Stanford, California, USA; 4 Department of Microbiology and Immunology, Stanford University School of Medicine, Stanford, California, USA; 5 Department of Pathology, University of Virginia, Charlottesville, Virginia, USA

## Abstract

**Background:**

*Entamoeba histolytica *is an intestinal protozoan parasite of humans. The genome has been sequenced, but the study of individual gene products has been hampered by the lack of the ability to generate gene knockouts. We chose to test the use of RNA interference to knock down gene expression in *Entamoeba histolytica*.

**Results:**

An episomal vector-based system, using the *E. histolytica *U6 promoter to drive expression of 29-basepair short hairpin RNAs, was developed to target protein-encoding genes in *E. histolytica*. The short hairpin RNAs successfully knocked down protein levels of all three unrelated genes tested with this system: Igl, the intermediate subunit of the galactose- and N-acetyl-D-galactosamine-inhibitable lectin; the transcription factor URE3-BP; and the membrane binding protein EhC2A. Igl levels were reduced by 72%, URE3-BP by 89%, and EhC2A by 97%.

**Conclusion:**

Use of the U6 promoter to drive expression of 29-basepair short hairpin RNAs is effective at knocking down protein expression for unrelated genes in *Entamoeba histolytica*, providing a useful tool for the study of this parasite.

## Background

The human parasite *Entamoeba histolytica *(*E. histolytica*) is a unicellular protozoal parasite that phylogenetically is placed on one of the lowermost branches of the eukaryotic tree, closest to *Dictyostelium discoideum *[1]. It is an unusual organism, having 9,938 predicted genes, with slightly less than one third (31.8%) of its predicted proteins having no homologues in GenBank [2]. Humans are its only natural hosts, and *E. histolytica *is spread by ingestion of contaminated food or water via the fecal-oral route and thus tends to endemically infect people under circumstances where hygiene is poor [3]. It has a simple life cycle, alternating between infective quadrinucleate cysts and invasive motile trophozoites [3]. 80% of people infected with *E. histolytica *are colonized asymptomatically; in the remaining 20%, trophozoites invade into the intestinal epithelium, resulting in clinical disease [3]. It is estimated that there are 50 million symptomatic cases of amebic colitis and 100,000 deaths per year worldwide due to *E. histolytica *[4].

The discovery that double-stranded RNA (dsRNA) can initiate post-transcriptional sequence-specific gene silencing of cellular genes [5] via translational repression or degradation of mRNA in most eukaryotic cells has become an important tool in assessing and manipulating gene function. This mechanism of RNA interference (RNAi) may have evolved as a defense against viruses and transposable elements with dsRNA intermediates [6,7]. The small RNA intermediates in this process, short interfering RNAs (siRNAs), result from dsRNA being cleaved at 21- to 23- nucleotide intervals [8] by an RNase III-type protein, Dicer [9], and are then incorporated into the RNA-induced silencing complex (RISC), which includes Argonaute "Slicer" protein [8,10]. The antisense strand of the siRNA is used to guide the RISC to its target mRNA, which is then cleaved by Argonaute [11,12]. RNAi effects can be amplified by the action of RNA-dependent RNA polymerases (RdRPs). siRNAs act as primers for RdRPs, which form new dsRNAs using the target mRNA as a template, which are subsequently cleaved into siRNAs with sequences corresponding to target mRNAs but differing from the original dsRNAs [13,14]. Genes encoding RdRPs have been identified in many organisms, but not in flies or mammals [12].

*E. histolytica *possesses the molecular machinery for RNAi. It has a gene [GenBank:XM_645408] [2,15,16] encoding a protein which has a single RNase III domain and possesses RNase III activity, and could perform the Dicer role as a dimer. It also has two Argonaute homologs [GenBank:XM_651344, XM_651422] [2,15-17] and an RdRP [GenBank:XM_646217] [2,15]. Exploitation of RNAi for knockdown of gene expression is an attractive approach for *E. histolytica*, as there is no evidence for meiotic division or detectable homologous recombination of genes [18-20], thus it has not been possible to generate gene knockouts [18,21]. Multiple copies of the genome, and even nuclei, occur in the parasite due to an apparent lack of the normal cell cycle regulatory checkpoints [22,23]. Homologous *Entamoeba *cell-cycle regulation genes are divergent from typical eukaryotic versions and may not have equivalent function [19]. This presents difficulties in studying gene function or in isolating recessive mutations [18]. The study of the function of individual genes in the past has been limited to other techniques, such as the over-expression of wild-type or mutant genes, and other methods of gene inactivation such as antisense [21,24]. Methods of RNAi used in *E. histolytica *have included the use of long dsRNA expressed by an *E. histolytica *RNA polymerase II promoter, which was successfully used to knock down expression of the *E. histolytica *proteins Diaphanous, Klp5 and EhSTIRP [18,25,26], and the soaking of trophozoites in artificial siRNAs to knock down γ-tubulin expression [20]. These reports of RNAi use in *E. histolytica *showed knockdown of a single gene or of a gene family. Here, we report in this study the success of the method of expression of short hairpin RNAs driven by the *E. histolytica *U6 promoter to knock down protein expression in *E. histolytica *of three unrelated genes.

Short hairpin RNAs (shRNAs) have a similar structure to siRNAs except the sense and antisense strands are connected at one end by a short loop, and function like siRNAs to knock down gene expression [27]. shRNAs can be produced from an expression vector as a single transcript from a RNA polymerase III promoter. The eukaryotic U6 promoter offers two advantages over other RNA polymerase III promoters: the promoter region immediately upstream of the transcribed sequence for the U6 small nuclear RNA gene includes all the required regulatory elements [28,29], and the termination sequence consists of 4 to 5 thymidine residues rather than a poly-A tail [28,29]. A variety of shRNA loop and stem lengths have been tested, with the loop UUCAAGAGA [28] used in a number of mammalian shRNA constructs, including Gou et al (2003) [30], and is also used in the constructs in this study. Longer hairpins with 29-base pair stems appear to be better inhibitors of gene expression than ones with shorter 19–21 bp stems [31]. Increased effectiveness has also been seen for similarly-sized longer artificial siRNAs, with only one siRNA apparently generated per longer shRNA or siRNA [31,32].

Genes selected for knockdown: The three genes selected for knockdown in this study, Igl, URE3-BP, and EhC2A, are genes involved in amebic virulence under study in our laboratory; they were selected since we wanted to create an additional tool for studying the function and role of these genes in amebic virulence.

Igl, the intermediate subunit of the galactose- and N-acetyl-D-galactosamine- (Gal/GalNAc) inhibitable lectin [33,34], is a 150 kDa protein. The Gal/GalNAc lectin, the major defined amebic adhesin, is a virulence factor mediating adherence to target cells in the first step of contact-dependent cell killing [3]. The lectin binds to terminal galactose or GalNAc residues in glycoproteins on the surfaces of target cells, and is composed of three subunits: the heavy subunit Hgl (containing a carbohydrate-recognition domain), the intermediate subunit Igl, and the light subunit Lgl [3]. The integral-membrane Hgl is disulfide-bonded to the GPI (glycosylphosphatidylinositol)-anchored Lgl. Igl is also GPI-anchored to the membrane [3]. Evidence that Igl is associated non-covalently with the Hgl-Lgl heterodimer includes that Igl and the Hgl-Lgl heterodimer co-migrate in native gel electrophoresis, and affinity-purification of Igl with monoclonal antibodies results in the co-purification of the Hgl-Lgl heterodimer [3,33,34]. Igl is encoded by two unlinked gene copies, Igl1 [GenBank:AF337950] [34] and Igl2 [GenBank:XM_647302] [2]; [GenBank:AF337951] [34], producing ~1100 aa proteins that are 81% identical and contain 32 CXXC repeats. CXXC repeats are also found in a family of transmembrane kinases of *E. histolytica *and the *Giardia lamblia *variant-specific surface proteins [35].

URE3-BP, Upstream Regulatory Element 3-Binding Protein [GenBank:AF291721] [36], is a 22.6 kDa calcium-regulated transcription factor encoding two EF-hand motifs, which are associated with calcium-binding activity [36]. URE3-BP binds to the URE3 (Upstream Regulatory Element 3) consensus motif, TATTCTATT, found in the promoter of *hgl5*, which is one of the genes encoding the Gal/GalNAc lectin heavy subunit, and is also present in the ferredoxin 1 (*fdx1*) promoter, thereby regulating the expression of these genes [36]. The human neuronal protein DREAM (calsenilin) is the only other known example of a calcium-responsive transcription factor with EF hands [36].

EhC2A [GenBank:XM_650207] [2] is a 22 kDa calcium-binding membrane protein containing a conserved C2 domain, is associated with the ability to bind phospholipids, and has a proline-rich C-terminal tail. This protein was found to be associated to the amebic phagosome [37]. A C2 domain, identified originally in protein kinase C, is a Ca^2+^-binding motif that allows calcium-dependent protein anchoring to or interaction with membranes; these domains are found in a number of signaling proteins in eukaryotes [38].

A gene for which we have previously shown knockdown is PATMK, Phagosome-Associated Transmembrane Kinase 96 [GenBank:XM_650501] [2,39]. PATMK is a transmembrane kinase family member found in the early phagosome and is involved in the phagocytosis of human erythrocytes [39]. It contains an intracellular putative kinase domain, a short membrane-spanning region, and an ectodomain containing CXXC-repeats like Igl [35,39].

We report here the effectiveness of shRNAs in silencing genes in *Entamoeba histolytica*. Expression of 29-bp shRNAs driven by the *E. histolytica *U6 promoter was successful in knocking down protein expression of the three different and unrelated genes in *E. histolytica *reported in this study, and we previously showed knockdown for a fourth gene [39]. This method of gene knockdown appears to function well for a variety of gene types, and should be a useful tool for studying gene function in this organism.

## Results

### Construction of shRNA constructs

**Figure 1:**
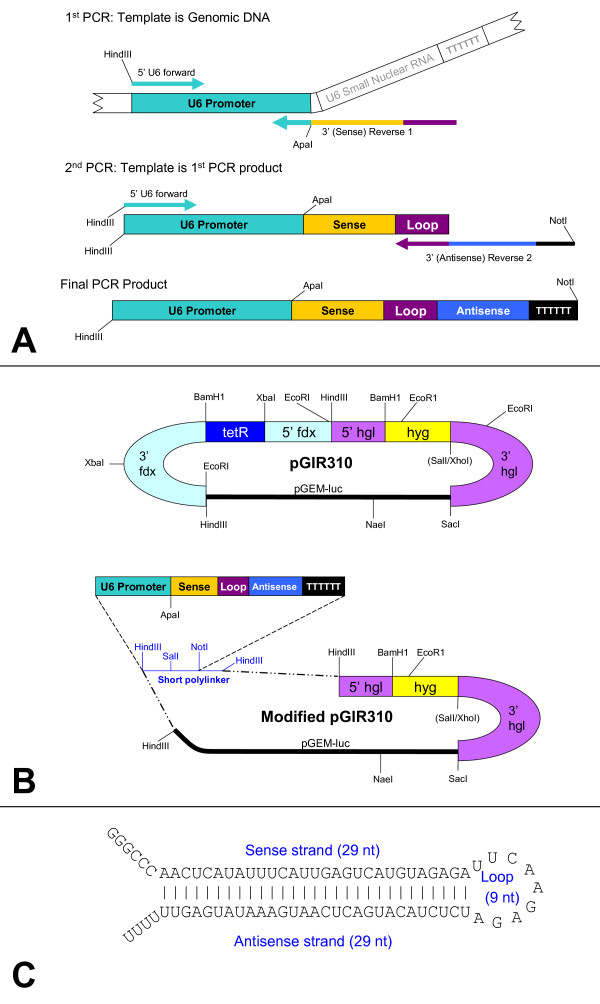
**shRNA system for *Entamoeba histolytica***. **(A)** Diagram of the two-step PCR process for generating short hairpins shRNA constructs were made using the method of Gou et al (2003) [30]. Genomic DNA (or subsequently, the cloned U6 promoter) was used as a template to amplify the *E. histolytica *U6 promoter and to add the hairpins. The primers in the first PCR round were the forward primer, containing a *Hin*dIII site and 5' end of the U6 promoter, and a first reverse primer, containing the U6 promoter 3' end, the shRNA sense strand sequence, and the 9-nucleotide loop. To yield the final product, in the second PCR round, the same forward primer was used, with a second reverse primer containing the loop sequence, the antisense strand sequence, the termination sequence, and a *Not*I recognition site, using the first round product as a template. The primers used to generate the PCR products are listed in Table 2. **(B) **Modification of amebic expression vector pGIR310 to express shRNA The tetracycline repressor cassette in expression vector pGIR310, a modification of pGIR308 [49,50], was replaced with a polylinker containing a *Sal*I and *Not*I site, flanked by *Hin*dIII sites. PCR products were cloned into the *Hin*dIII and *Not*I sites. pGIR310 confers hygromycin resistance in amebae and ampicillin resistance in *E. coli *bacteria. **(C)** Expected structure of 29-basepair shRNA before processing by Dicer The 29-basepair stem and 9-nucleotide loop are shown.

The RNA polymerase III promoter of the *E. histolytica *U6 gene [GenBank:U43841] [40] was amplified beginning at -333 from the transcription start site of the U6 small nuclear RNA gene, and the shRNA-encoding DNA was added by PCR at the transcription start site [30,39] (Figure 1A). The resulting U6 promoter-shRNA constructs were cloned into pGIR310 modified to contain a short polylinker (Figure 1B). The shRNAs were designed to have a 29-nucleotide complementary stem with a 9-nucleotide loop (Figure 1C). The sense strand sequences of the shRNA constructs transfected into HM1:IMSS trophozoites, the oligonucleotide (oligo) sequences used to create them by PCR, and the oligo sequences used in quantitative reverse-transcription real-time PCR (qRT-PCR) amplification to assess mRNA knockdown are shown in Tables 1, 2, 3.

**Table 1:** Sequences chosen to generate shRNA constructs that were successfully transfected into amebae

Name	Sequence	Location in mRNA/cDNA (bp from ATG)	Total length of target mRNA (bp)
Igl1 (272–300)	AAGTAAATACATCATCACACTCTGGAAAT	272–300 (Igl1)	3306 (Igl1), 3318 (Igl2)
Igl (1198–1226)	AATGGACTTACATTGAATGGAACTCATTG	1198–1226 (Igl1)	
Igl (2412–2440)	AACAGAATGTTCAGATGGTTTTAGTGGAC	2412–2440 (Igl1)	
Igl (2777–2805)	AAGGAACATGTATACCATGCACATCACCA	2777–2805 (Igl1)	
URE3-BP (350–378)	AACTTGCATACAATCTCTTCGTTATGAAC	350–378	663
URE3-BP (580–608)	AATCCATACTATGGTCCAATGAAACCATT	580–608	
EhC2A (363–391)	AATGGTTCCACCAATGCAACCAGGCATGA	363–391	567
EhC2A (502–530)	GCTTACCCACCACCTGGATATCCACCAA	502–530; also 406–434	
EhC2A (363–391 scrambled)	AAGGCTAGACAATCCAGACCGTTCCAGAT	Does not match any *E. histolytica *mRNA	None

GFP	AAGGTGATGCAACATACGGAAAAC	Does not match any *E. histolytica *mRNA	None

The Ambion siRNA finder [51] was used to select 21 mers from the entire coding sequence of URE3-BP, the poly-proline region of EhC2A, or the identical or divergent regions of Igl1 and Igl2, which were then checked for sufficient GC content, lengthened to 29 nucleotides, and tested for sufficient sequence uniqueness by blasting each 29 mer using the *E. histolytica *Genome Project database [52]. A scrambled sequence was created as a control for EhC2A. A sequence directed against GFP [30] was included as a control for the Igl and URE3-BP selections. The constructs are named such that the numbers in parentheses following the gene name indicated the location of the shRNA sense strand within that gene sequence.

**Table 2:** Oligos used for generating shRNA constructs by PCR and transfected into amebae

Oligo Name	Oligo Sequence
U6 *Hin*dIII forward	CTACTGAAGCTTGTTTTTATGAAAAAGTGTATTTGC
GFP R1	TCTCTTGAAGTTTTCCGTATGTTGCATCACCTTGGGCCCAATTTTATTTTTCTTTTTATCC
GFP R2	TCGATCGCGGCCGCAAAAAAGGTGATGCAACATACGGAAAACTCTCTTGAA
Igl1 (272–300) R1	TCTCTTGAAATTTCCAGAGTGTGATGATGTATTTACTTGGGCCCAATTTTATTTTTCTTTTTATCC
Igl1 (272–300) R2	TCGATCGCGGCCGCAAAAAAGTAAATACATCATCACACTCTGGAAATTCTCTTGAA
Igl (1198–1226) R1	TCTCTTGAACAATGAGTTCCATTCAATGTAAGTCCATTGGGCCCAATTTTATTTTTCTTTTTATCC
Igl (1198–1226) R2	TCGATCGCGGCCGCAAAAAATGGACTTACATTGAATGGAACTCATTGTCTCTTGAA
Igl (2412–2440) R1	TCTCTTGAAGTCCACTAAAACCATCTGAACATTCTGTTGGGCCCAATTTTATTTTTCTTTTTATCC
Igl (2412–2440) R2	TCGATCGCGGCCGCAAAAAACAGAATGTTCAGATGGTTTTAGTGGACTCTCTTGAA
Igl (2777–2805) R1	TCTCTTGAATGGTGATGTGCATGGTATACATGTTCCTTGGGCCCAATTTTATTTTTCTTTTTATCC
Igl (2777–2805) R2	TCGATCGCGGCCGCAAAAAAGGAACATGTATACCATGCACATCACCATCTCTTGAA
URE3-BP (350–378) R1	TCTCTTGAAGTTCATAACGAAGAGATTGTATGCAAGTTGGGCCCAATTTTATTTTTCTTTTTATCC
URE3-BP (350–378) R2	TCGATCGCGGCCGCAAAAAACTTGCATACAATCTCTTCGTTATGAACTCTCTTGAA
URE3-BP (580–608) R1	TCTCTTGAAAATGGTTTCATTGGACCATAGTATGGATTGGGCCCAATTTTATTTTTCTTTTTATCC
URE3-BP (580–608) R2	TCGATCGCGGCCGCAAAAAATCCATACTATGGTCCAATGAAACCATTTCTCTTGAA
EhC2A (363–391) R1	TCTCTTGAATCATGCCTGGTTGCATTGGTGGAACCATTGGGCCCAATTTTATTTTTCTTTTTATCC
EhC2A (363–391) R2	TCGATCGCGGCCGCAAAAAATGGTTCCACCAATGCAACCAGGCATGATCTCTTGAA
EhC2A (502–530) R1	TCTCTTGAAATTGGTGGATATCCAGGTGGTGGGTAAGCGGGCCCAATTTTATTTTTCTTTTTATCC
EhC2A (502–530) R2	TCGATCGCGGCCGCAAAAAAGCTTACCCACCACCTGGATATCCACCAATTCTCTTGAA
EhC2A (363–391 scrambled) R1	TCTCTTGAAATCTGGAACGGTCTGGATTGTCTAGCCTTGGGCCCAATTTTATTTTTCTTTTTATCC

EhC2A (363–391 scrambled) R2	TCGATCGCGGCCGCAAAAAAGGCTAGACAATCCAGACCGTTCCAGATTCTCTTGAA

The sequences shown in Table 1 were used to design primers for two-step PCR, based on the method used by Gou et al (2003) [30] and diagrammed in Figure 1A. The final PCR product contained the *E. histolytica *U6 promoter with a *Hin*dIII site on the 5' end, an *Apa*I site at the 3' end of the U6 promoter, the 29-nt sense strand of the hairpin, the 9 bp loop TTCAAGAGA, the antisense strand of the hairpin, and the U6 terminator sequence followed by a *Not*I restriction site. The forward primer, "U6 *Hin*dIII forward", contained the *Hin*dIII recognition site and the 5' end of the U6 promoter, the first reverse primer (R1) contained the sequence of the sense strand of the shRNA and the future loop, and the second reverse primer (R2) contained the loop sequence, the antisense strand sequence, and the U6 termination sequence. A control GFP sequence [30] was used to design oligos for creating a shRNA construct as a transfection control.

**Table 3:** Sequences of oligos used for amplification in qRT-PCR

Oligo Name	Oligo Sequence	mRNA/cDNA section amplified (bp from ATG)	Total length of mRNA (bp)
Igl 5' F	GCTGTTCCACATTGTGCATCAGTTTCAAATG	85–450 (Igl1), 85–459 (Igl2)	3306 (Igl1), 3318 (Igl2)
Igl 5' R	TTCTGCATGATCTTCTGTAGTTGCATTATCACATAAC		
Igl 3' F	TGAAGGCACTTCTACAGAAGATAATAAAAT	2967–3166 (Igl1), 2979–3178 (Igl2)	
Igl 3' R	TATGTCTTGAACATGGAATACATGATC		
Igl1 F	TCTTGTAATAAGTTCCCGGAGCA	634–841 (Igl1)	
Igl1 R	CATCAGAAACAGTACATCTTTTATTACATG		
Igl2 F	GTACTAAATACCCAGATCATTGTTCAAA	643–841 (Igl2)	
Igl2 R	CATCAGAAACAGTACATCTTTTATTACATG		
URE3-BP 5' F	CCTGTAGCTAATTTCTGTTTATGGAATC	10–155	663
URE3-BP 5' R	CTTGTATATTGATCTAATGGGATAGTGTTAAG		
URE3-BP Middle F	GATGAGAATTTTTGATACTGATTTTAATGGAC	276–454	
URE3-BP Middle R	GATTAATATAGAATCCAAGTTGTTGAAGAG		
URE3-BP 3' F	CTGTGATCTTAATTGTTGGATTG	504–658	
URE3-BP 3' R	CCAAGAGGGAAGTAACAACGT		
Actin F	GCACTTGTTGTAGATAATGGATCAGGAATG	variable (detects all family members/alleles)	variable
Actin R	ACCCATACCAGCCATAACTGAAACG		
Jacob F	CAAAGGAGTTCAAATGGGATGTGTTAG	variable (detects all family members/alleles)	variable

Jacob R	TTATTTGGTGTAGGAGTTGGTAATGGG		

Oligo pairs were designed to amplify short sections of Igl or URE3-BP. For Igl, four pairs of oligos were used: one amplifying the 5' end (Igl 5' oligo pair) and one the 3' end (Igl 3' oligo pair) of Igl1 and Igl2 simultaneously; and a pair each to amplify a short section unique to Igl1 or Igl2 (Igl1 oligo pair and Igl2 oligo pair, which have the same reverse primer in common) near the 5' end of the mRNA. Three oligo pairs were used to amplify short sections of URE3-BP: one pair the 5' end, one pair the middle, and one pair the 3' end. The actin and Jacob primers were designed to amplify all family members or alleles [35].

### shRNA transfectants

Transfectants were maintained at 15 μg/ml hygromycin. For knockdown studies, the hygromycin concentration was increased every 24 hours until the final level of selection was achieved, and was maintained for 48 hours, in order to increase the copy number of the episomal shRNA vector [41-43]. The level of hygromycin selection was increased until the desired knockdown was attained, up to 100 μg/ml. Transfected trophozoites selected with 100 μg/ml hygromycin continued to grow and divide for at least two weeks under continuous selection. A shRNA directed against green fluorescent protein (GFP) [30], with a sequence matching nothing in the *E. histolytica *genome, was utilized as a control for transfection and hygromycin selection for the Igl and URE3-BP transfectants. GFP shRNA transfectants were selected with the same level of hygromycin as other shRNA transfectants. For EhC2A, a scrambled control matching nothing in the *E. histolytica *genome was created, containing the same nucleotides as the EhC2A (363–391) shRNA, but in a different order. Sequences of the shRNA sense strands are shown in Table 1. Non-transfected HM1-IMSS amebae were also included, with the results for Western blotting and qRT-PCR being statistically the same as the GFP controls. Three biological replicates were grown per shRNA transfectant, and one for the nontransfected HM1:IMSS amebae. All sample trophozoites were grown in 25 cm^2 ^tissue culture flasks, and were harvested for crude lysate and for RNA isolation on the same day from the same flask. For protein and mRNA comparison, actin was used as the "housekeeping" control gene, as a loading and normalization control.

### Knockdown of Igl protein

**Figure 2:**
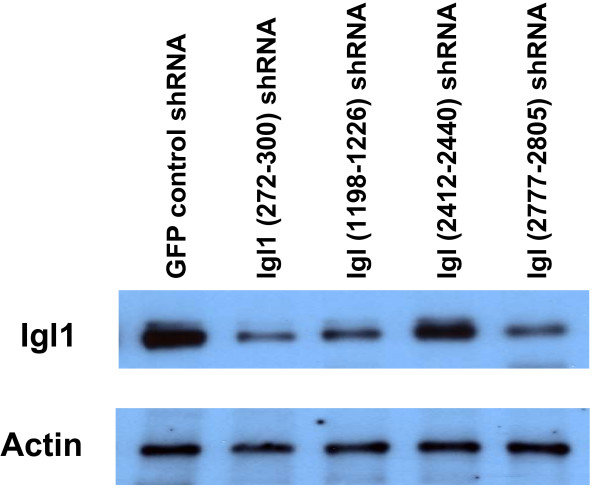
**Western blot for Igl shRNA transfectants**. A representative Western blot is shown with one biological replicate each for the GFP control shRNA transfectant, the Igl1-specific (272–300), the Igl (1198–1226), the Igl (2412–2440), and the Igl (2777–2805) shRNA transfectants. HM1:IMSS samples are not shown. Results shown are representative of three biological replicates per shRNA transfectant with each sample run in triplicate. Serial dilutions of the crude lysates (1:2, 1:4, and 1:8) were also performed for each sample. Each membrane was probed with anti-actin antibody as a loading control, or with anti-Igl1 antibody. Igl1 protein levels for the Igl shRNA and GFP shRNA transfectants and HM1:IMSS nontransfected amebae are summarized in Table 4.

Four Igl shRNA constructs targeted Igl. One construct, Igl1 (272–300), specifically targeted Igl1. Three constructs, Igl (1198–1226), Igl (2412–2440), and Igl (2777–2805), were targeted to sequences conserved in both Igl1 and 2 (Table 1). The GFP shRNA transfectants were used as controls. Transfected trophozoites were selected with 100 μg/ml hygromycin for 48 hours before harvesting. Blots were probed with anti-Igl1 antibody, and with anti-actin antibody as a loading and normalization control. The level of Igl1 in the GFP shRNA transfectants was defined to be 100% (Figure 2, Table 4). The Igl1-specific (272–300) shRNA transfectant had a decreased amount of Igl1 protein, 27.8 ± 3.9%, as compared to the GFP shRNA control (Figure 2, Table 4). Igl (1198–1226) had 42.3 ± 6.2% and Igl (2777–2805) had 38.1 ± 9.4% of the GFP control Igl1 level. The Igl (2412–2440) shRNA construct had no effect on Igl1 levels (95.3 ± 9.7% of the level in the GFP shRNA transfectants) (Figure 2, Table 4). HM1:IMSS nontransfected amebae were not statistically different from the GFP shRNA control (Table 4). The Igl (1198–1226) and Igl (2777–2805) transfectants, when selected with 30 μg/ml hygromycin rather than 100 μg/ml, yielded less knockdown, having ~70% and ~65% of the control level of Igl1 (data not shown).

**Table 4:** Summary of Igl1 protein levels in Igl shRNA transfectants

shRNA Transfectant or Control Sample	% of Igl1 protein level (± SE)	P-value
GFP	100.0 ± 3.6	
HM1:IMSS	115.5 ± 11.8	0.1449
Igl (2412–2440)	95.3 ± 3.2	0.2078
Igl1 (272–300)	27.8 ± 1.3	< 0.0001
Igl (1198–1226)	42.3 ± 2.1	< 0.0001

Igl (2777–2805)	38.1 ± 3.1	< 0.0001

The average level of Igl1 protein in the GFP control shRNA transfectants was defined as 100% expression of Igl1 protein for computational purposes. Protein levels of Igl1, using actin as a standard for comparison, were quantified from Western blotting. Values are expressed as the percentage of the GFP level ± SE, with the P-value following each, calculated using Student's t test. For Western blotting, there were three biological replicates, each run in triplicate sets of serial dilutions (1:2, 1:4, and 1:8), with the exception of the HM1:IMSS nontransfected samples having one biological replicate rather than three. Protein levels were not statistically different between the Igl1 (272–300), Igl (1198–1226), and Igl (2777–2805) samples (tested with ANOVA, one-tailed, α = 0.05, 0.1 < P < 0.25) or the GFP, HM1:IMSS, and Igl (2412–2440) samples (tested with ANOVA, one-tailed, α = 0.05, P > 0.25). A representative Western blot is shown in Figure 2.

### Knockdown of Igl mRNA

Short sections of Igl were amplified via qRT-PCR using template cDNAs synthesized from the Igl and control GFP shRNA transfectant mRNAs. Four oligo pairs were used to amplify Igl. Two sets of oligos targeted both Igl1 and Igl2 simultaneously, with one pair amplifying a 5' section and the other a 3' section conserved in both Igl1 and Igl2. The two others were specific for Igl1 or Igl2, targeting a non-conserved region. The oligo sequences and regions of Igl transcript amplification are shown in Table 3, and summarized qRT-PCR data for Igl is shown in Table 5. All samples were compared to the GFP control shRNA transfectants. Three of the four Igl shRNA transfectants showed knockdown of Igl transcripts for all sets of oligo pairs, ranging between ~60 and ~80% of the Igl level in the GFP shRNA control (Table 5). Igl (2412–2440) shRNA transfectants did not show any knockdown, and the HM1:IMSS nontransfected trophozoites were not statistically different from the GFP shRNA control (Table 5).

**Table 5:** Summary of Igl mRNA levels in Igl shRNA transfectants

shRNA transfectant or control sample	Igl 5' oligo pair	P-value	Igl 3' oligo pair	P-value	Igl1 oligo pair	P-value	Igl2 oligo pair	P-value
GFP6	100.0 ± 4.1	--	100.0 ± 4.9	--	100.0 ± 3.0	--	100.0 ± 4.0	--
HM1:IMSS	101.4 ± 4.3	0.7741	96.1 ± 3.5	0.3239	105.5 ± 3.1	0.1382	103.9 ± 6.1	0.5713
Igl (2412–2440)	100.6 ± 5.0	0.9172	103.4 ± 9.1	0.7717	91.1 ± 6.9	0.2426	106.0 ± 5.2	0.2919
Igl1 (272–300)	71.3 ± 2.9	<0.0001	67.1 ± 3.0	<0.0001	61.1 ± 3.2	<0.0001	70.2 ± 2.7	<0.0001
Igl (1198–1226)	70.9 ± 2.7	<0.0001	62.1 ± 1.6	<0.0001	68.3 ± 2.5	<0.0001	76.8 ± 1.6	<0.0001

Igl (2777–2805)	68.1 ± 3.3	<0.0001	62.3 ± 2.9	<0.0001	74.1 ± 3.3	<0.0001	77.8 ± 3.0	<0.0001

For qRT-PCR, samples were amplified with the actin oligo pair as a control, or with four pairs of Igl oligos: Igl 5', amplifying the 5' end of both Igl1 and Igl2, Igl 3', amplifying both Igl1 and Igl2 at the 3' end, and oligos specific for Igl1 and Igl2 individually, amplifying Igl1- or Igl2-specific sequences near the 5' end. Oligo sequences are shown in Table 3. Three biological replicates were each assayed in quadruplicate sets with each oligo pair, with the exception of the HM1:IMSS samples, which had one biological replicate. Igl and actin levels were calculated by using both the relative standard curve and the ΔΔC(t) method [54,55] and actin was used as the normalization control. The average level of Igl in the GFP control shRNA transfectants was defined as 100% expression of Igl mRNA for computational purposes. Igl levels in the Igl transfectant samples and nontransfected HM1:IMSS were compared to the GFP control, and are shown as the percentage of Igl mRNA relative to the GFP control (± SE). Statistical analysis was performed using Student's t test (two-tailed), groups were compared using ANOVA, and the GraphPad QuickCalcs P-value calculator [53] was used to calculate P-values.

### Knockdown of URE3-BP protein

**Figure 3:**
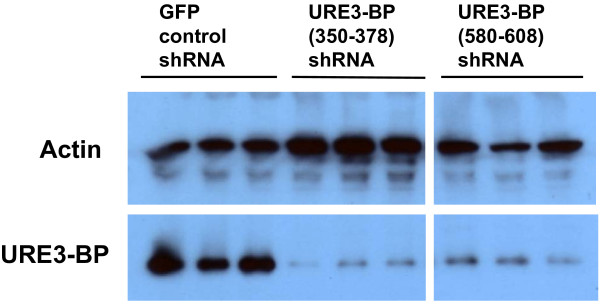
**Western blot for URE3-BP shRNA transfectants**. A representative Western blot is shown with three biological replicates each (one dilution shown) for GFP control, URE3-BP (350–378), and URE3-BP (580–608) shRNA transfectants. HM1:IMSS samples are not shown. Results are representative of three biological replicates per shRNA transfectant with each sample run in triplicate. Serial dilutions of the crude lysates (1:2, 1:4, and 1:8) were also done for each sample. Each membrane was probed with anti-actin antibody as a loading control, or with anti-URE3-BP antibody. URE3-BP protein levels are summarized in Table 6.

Two shRNA constructs were used to target URE3-BP: URE3-BP (350–378) and URE3-BP (580–608). Transfected trophozoites were selected with 100 μg/ml hygromycin (GFP control or URE3-BP (350–378) shRNA) or 75 μg/ml hygromycin (URE3-BP (580–608) shRNA) for 48 hours before harvesting. Actin was used as a normalization and loading control. There was significant reduction of URE3-BP protein in both URE3-BP shRNA transfectants: for URE3-BP (350–378) it was 10.8 ± 1.0% and 13.8 ± 2.6% for URE3-BP (580–608) as compared to the GFP shRNA control (Figure 3, Table 6). HM1:IMSS samples were also included, but were not statistically different from the GFP shRNA control (Table 6).

**Table 6:** Summary of URE3-BP protein levels in URE3-BP shRNA transfectants

shRNA transfectant or control sample	% of control protein level (± SE)	P-value
GFP	100 ± 9.9	--
HM1:IMSS	111.3 ± 15.8	0.6189
URE3-BP (350–378)	10.8 ± 1.0	<0.0001

URE3-BP (580–608)	13.8 ± 2.6	<0.0001

The average level of URE3-BP protein was defined as being 100% in the GFP shRNA control transfectants. The levels of URE3-BP and the actin standard were quantified from Western blotting. Values are expressed as the percentage of URE3-BP protein or mRNA of the GFP control shRNA transfectant level ± SE, with the P-value following each. There were three biological replicates for all samples, each run in triplicate plus serial dilutions (1:2, 1:4, and 1:8), except for HM1:IMSS nontransfectants, which had one biological replicate run in triplicate plus serial dilutions. All samples were normalized to actin, and compared to the GFP control using Student's t test. URE3-BP protein levels are not statistically different between the URE3-BP (350–378) and (580–608) samples (two-tailed Student's t test for comparing two sample averages, P = 0.3262) or between the GFP and HM1:IMSS nontransfected samples (two-tailed Student's t test for comparing two sample averages, P = 0.2346). A representative Western blot is shown in Figure 3.

### Knockdown of URE3-BP mRNA

Three different oligo pairs, one amplifying the 5' end of URE3-BP, one the 3' end, and one a section in the middle, were used in qRT-PCR to amplify URE3-BP in cDNA from GFP shRNA control transfectants, URE3-BP (350–378) and URE3-BP (580–608) shRNA transfectants, and HM1:IMSS nontransfected trophozoites. Oligo sequences are shown in Table 3. Actin was used as the normalization control. The URE3-BP (350–378) shRNA transfectant had an average of about 69% of the GFP control URE3-BP transcript level, and the URE3-BP (580–608) shRNA transfectant had about 13% of the of the GFP shRNA control URE3-BP level (Table 7).

**Table 7:** Summary of mRNA levels in GFP shRNA control transfectants, URE3-BP shRNA transfectants, and nontransfected HM1:IMSS trophozoites

shRNA transfectant or control sample	URE3-BP 5' oligo pair	P-value	URE3-BP middle oligo pair	P-value	URE3-BP 3' oligo pair	P-value
GFP	100.0 ± 2.9	--	100 ± 2.8	--	100 ± 4.3	--
HM1:IMSS	106.4 ± 5.8	0.2928	108.9 ± 5.6	0.1008	102.8 ± 5.0	0.5792
URE3-BP (350–378)	67.0 ± 2.5	<0.0001	67.4 ± 2.0	<0.0001	72.2 ± 2.8	<0.0001

URE3-BP (580–608)	12.4 ± 0.8	<0.0001	13.5 ± 3.3	<0.0001	12.5 ± 3.8	<0.0001

The average URE3-BP transcript level as measured by qRT-PCR and normalized to actin was defined as being 100% in the GFP shRNA control transfectants. HM1:IMSS nontransfected amebae were also included. Three different oligo pairs amplifying the 5', middle, and 3' sections of URE3-BP were used (sequences and locations are shown in Table 3). Student's t test was used for statistical analysis. Three biological replicates were each assayed in quadruplicate with each oligo pair, with the exception of the HM1:IMSS samples, which had one biological replicate. Values are expressed as the percentage of URE3-BP mRNA of the GFP control shRNA transfectant level ± SE, with the P-value following each.

### Knockdown of EhC2A protein

**Figure 4:**
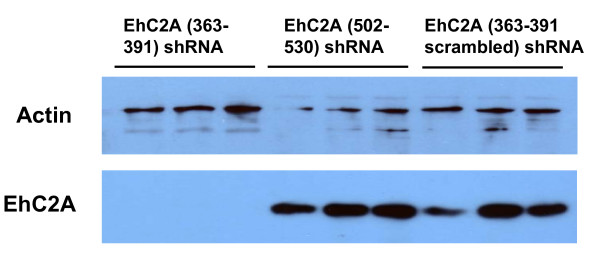
**Western blot for EhC2A transfectants**. A representative Western blot is shown with three biological replicates each for EhC2A (363–391), EhC2A (502–530), and EhC2A (363–391 scrambled control) shRNA transfectants. Results are representative of three biological replicates per shRNA transfectant with each sample run in triplicate. Each sample was also serially diluted 1:2, 1:4, and 1:8. Each membrane was probed anti-EhC2A and with anti-actin antibody as a loading control. The level of EhC2A protein in the scrambled control transfectant was defined as 100% (± 5%). The EhC2A (363–391) shRNA transfectant had strongly reduced levels of EhC2A protein: it was only 3.0 ± 0.4% of the scrambled control. The EhC2A (502–530) shRNA transfectant had no knockdown effect on EhC2A levels (106.1 ± 7.3%).

Two shRNA constructs targeted EhC2A, EhC2A (363–391) and EhC2A (502–530). Transfectants were selected with 90 μg/ml hygromycin for 48 hours before harvesting. The scrambled control EhC2A (363–391 scrambled) shRNA transfectant was used as a control for EhC2A protein levels. HM1:IMSS nontransfected amebae were not included. The level of EhC2A protein in the EhC2A (363–391 scrambled) control shRNA transfectant was defined as 100 ± 5.0% (± SE). The EhC2A (363–391) shRNA transfectant yielded a knockdown of EhC2A protein to a level of 3.0 ± 0.4% (P < 0.0001). The EhC2A (502–530) shRNA transfectant had no knockdown effect on EhC2A levels (106.1 ± 7.3%) and was statistically the same (P = 0.3141) as the EhC2A (363–391 scrambled) shRNA control transfectant (Figure 4). Student's t test was used for statistical analysis. qRT-PCR was not performed for these samples.

### Northern blots of small RNAs

**Figure 5:**
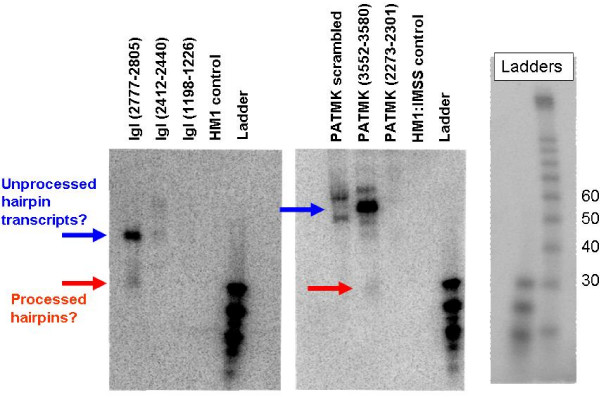
**Northern blots of small RNAs extracted from Igl and PATMK transfectants**. To test if the U6 promoter was driving hairpin expression, shRNA transfectants (PATMK (3552–3580), PATMK (2273–2301), PATMK (3552–3580 scrambled) [39], Igl (1198–1226), Igl (2412–2440), and Igl (2777–2805) were selected with 30 μg/ml hygromycin for 48 hours before harvesting. HM1:IMSS non-transfected amebae were included as negative controls. Small RNAs were extracted using the mirVana™ miRNA Isolation Kit (Ambion) (Applied Biosystems/Ambion, Austin, TX, USA). Fifty μg small RNA were loaded per lane on a 12% denaturing acrylamide gel and transferred to membrane. rRNA bands were analyzed to ensure equal RNA loading. Oligo probes matching to the sense and antisense strands of the hairpins were end-labeled with ^32^P and were hybridized with each corresponding sample blot overnight at 37°C overnight, washed with low and medium stringency conditions, and exposed overnight to film. Note the two product sizes, which may correspond to the unprocessed hairpin (~60–70 nucleotides) (blue arrows) and the processed siRNA products (~30 nucleotides) (red arrows).

Since the *E. histolytica *U6 promoter had never been characterized, we tested if shRNAs or other small RNAs were being produced by the U6 promoter. The PATMK samples were included because they had been shown to have significant knockdown of PATMK protein levels as compared to the scrambled PATMK shRNA control transfectant [39], and therefore would be good candidates for expressing the shRNAs. Northern blotting of the PATMK [39] and Igl shRNA transfectant small RNAs was performed. Transfected trophozoites were selected with 30 μg/ml hygromycin for 48 hours before harvesting, since we had seen protein knockdown previously at that level of selection [39]. Non-transfected HM1:IMSS amebae were included as a negative control. Fifty μg of small RNAs from PATMK shRNA transfectants [39] and the Igl shRNA transfectants were probed with oligo probes targeting the respective sense and antisense strands of the shRNAs (Figure 5). The PATMK (3552–3580) [39] and Igl (2777–2805) shRNA samples had substantial expression of ~70 and ~30 nucleotide small RNAs, the expected sizes for the unprocessed hairpin and the processed siRNA respectively. There was not a correlation of the small RNA abundance with the degree of protein knockdown, as the small RNAs were more abundant in Igl (2777–2805) than in Igl (1198–1226), yet both had similar degrees of knockdown.

## Discussion

We have utilized the U6 promoter to drive expression of shRNAs with a 29-bp stem and a 9-nt loop to knock down protein expression of three unrelated genes: a membrane protein, Igl, the intermediate subunit of the Gal/GalNAc lectin; URE3-BP, a calcium-regulated transcription factor, upstream regulatory element 3- binding protein; and EhC2A, a membrane-binding protein. Previously we had reported preliminary experience with this system in the near-complete knockdown of phagosome-associated transmembrane kinase 96 (PATMK) [39]. In the work reported here, the highest level of protein knockdown for Igl was 72%, for URE3-BP 89%, and for EhC2A 97%. We concluded that this was a reliable and effective system for gene knockdown in *E. histolytica*. This method has advantages over other methods used for gene silencing: the U6-shRNA expression cassettes are small (420 bp), appear to be active against different types of genes, yield significant knockdown, and the expression vector, once transfected, allows continuous expression of shRNAs, thus avoiding performing multiple transfections, and the shRNAs can be easily synthesized via PCR.

Not every transfected shRNA construct was equally effective in silencing gene expression. For example, neither the EhC2A (502–530) nor the Igl (2412–2440) shRNA construct blocked gene expression. In the case of Igl (2412–2440), the run of four thymidines at positions 19–23 in the shRNA sense strand could possibly cause RNA polymerase III to terminate the transcript prematurely. In the case of the EhC2A (502–530) shRNA construct, the shRNA could bind to two locations in the mRNA, both the originally targeted region (502–530), but also about 100 nt upstream (406–434), and perhaps this had an inhibitory effect on the ability of this shRNA to allow knockdown.

Factors other than the shRNA sequence affect the ability of a shRNA to down-regulate gene expression. The secondary structure of the transcript affects the ability of the RISC to bind to its target site [44,45], and the relative abundance and stability of an mRNA may play a significant role in determining whether a given shRNA will effectively lead to the degradation of its target message. In addition, the stability of a protein product may also be a determinant in the detection of a knockdown phenotype. The protein with the least knockdown in these studies, Igl, was the most abundant; EhC2A was the least abundant and had the most knockdown [46]. The level of hygromycin utilized to select for transfectants was an important determinant of the extent of protein knockdown. Igl knockdown was twice as effective with 100 μg/ml as with 30 μg/ml of hygromycin selection.

The qRT-PCR data was not correlated directly with the level of protein knockdown. For the Igl transfectants, the mRNA knockdown level was not as high as the protein knockdown level, indicating the possibility that the protein could have a high turnover rate or be somewhat unstable. For URE3-BP, the URE3-BP (350–378) and (580–608) transfectants had similar levels of protein knockdown; however, the mRNA levels in the URE3-BP (350–378) transfectants were higher (67% of the control level), versus the URE3-BP (580–608) transfectants (13.5% of the control level). This difference is probably not due to partial mRNA decay, since the qRT-PCR data showed consistent URE3-BP levels among the three oligo pairs amplifying the 5', middle, and 3' sections of the transcript. One possible explanation could be that the secondary structure of the URE3-BP mRNA at the location of the URE3-BP (350–378) shRNA could interfere sufficiently with the RISC being able to cleave the mRNA but still allow RISC binding, allowing for a degree of translational inhibition in addition to some mRNA destruction.

The *E. histolytica *U6 promoter appears to be functional and producing shRNAs: the Northern blots of the small RNAs detected two sizes of small RNAs when probed with oligos that were complementary to the individual sense and antisense strands of the shRNAs. These may represent the unprocessed hairpin and the resulting siRNAs after Dicer processing. Surprisingly, the abundance of the small RNA was not proportional to the level of silencing. Northern blots may not be sensitive enough to identify low-level small RNA production, with low-level production adequate for protein knockdown.

## Conclusion

We report the knockdown of three genes in this study: Igl, the intermediate subunit of the Gal/GalNAc lectin; the calcium-responsive transcription factor URE3-BP; the membrane-binding protein EhC2A, by transfecting *E. histolytica *with expression vectors using the *E. histolytica *U6 promoter to drive expression of shRNAs targeting endogenous genes. We have previously reported the knockdown of transmembrane kinase PATMK [39]. These genes come from different families, with different functions, so this shRNA knockdown method appears robust and not specific to only one gene or gene family.

## Methods

### Culture of trophozoites

*E. histolytica *strain HM1:IMSS trophozoites were grown axenically in TYI-S-33 (Trypticase-yeast extract-iron-serum) (TYI) medium supplemented with 1× Diamond's vitamins (SAFC Biosciences, Lenexa, KS, USA), 15% heat-inactivated adult bovine serum (Gemini Bio-Products, West Sacramento, CA), 100 U of penicillin/ml and 100 μg streptomycin sulfate/ml (Gibco/Invitrogen, Carlsbad, CA, USA), at 37°C in 25 cm^2 ^tissue culture flasks [47] in a volume of 50 ml, and then transfected as described below.

### Transfection of amebae

Plasmid DNA was prepared using the HiSpeed Qiagen Maxi Kit (Qiagen, Valencia, CA, USA). Medium 199 (M199) (Gibco BRL/Invitrogen, Carlsbad, CA, USA) was supplemented with 5.7 mM cysteine, 25 mM HEPES, and 0.6 mM ascorbic acid [48], adjusted to pH 7.0 and filter-sterilized. Twenty μg plasmid DNA diluted in 100 μl supplemented M199s medium (M199S) in 2-ml microcentrifuge tubes was mixed with 15 μl of SuperFect or Attractene transfection reagent (Qiagen, Valencia, CA, USA), and incubated at room temperature to allow transfection-complex formation as per the manufacturer's instructions. Heat-inactivated bovine serum was added to the remaining M199S to a 15% concentration. Amebae were harvested by tapping the tissue culture flasks on a benchtop, were centrifuged at 200 × g for 5 min at 4°C, and suspended in M199S with serum to 2.5 × 10^5 ^amebae/ml. Tubes containing transfection complexes were filled with the suspended trophozoites, the contents mixed by inversion, and the tubes were incubated horizontally for 3 hours at 37°C. Tube contents were added to warm TYI in 25 cm^2 ^tissue culture flasks, and incubated overnight at 37°C. 15 μg/ml hygromycin (Invitrogen, Carlsbad, CA, USA) was added for selection after the overnight incubation [49]. After 4–5 days, 25 ml of the TYI was removed to a new 25 cm^2 ^tissue culture flask, and 25 ml fresh TYI with hygromycin was added to each of the flasks. Transfectants were usually apparent 1–2 weeks after transfection.

### *E. histolytica *shRNA constructs

All short hairpin RNAs used in this study were expressed by the U6 promoter [GenBank:U43841] [41] (Figure 1A) and cloned into the amebic expression vector pGIR310, a modification of pGIR308 [49,50] by the addition of a short polylinker containing *Hin*dIII, *Sal*I, and *Not*I restriction sites (Figure 1B). Modified pGIR310 conferred resistance to hygromycin in *E. histolytica *and to ampicillin in *Escherichia coli *(*E. coli*). All shRNA constructs used in these studies had the same structure: a short hairpin consisting of a 29-nucleotide sense strand, followed by the 9-nucleotide loop and the 29-nucleotide complementary antisense strand (Figure 1).

### Sequence selection for shRNA constructs

The Ambion siRNA finder [51] was used to select possible siRNA sequences of 21 mers beginning with two adenine residues. To select sequences that would target Igl1 and Igl2 both separately and simultaneously, those portions of their coding sequences which were identical or divergent were input separately, while the entire coding sequence of URE3-BP was used to select siRNA sequences. For EhC2A the portion of the gene sequence selected for targeting was the poly-proline region (bases 301–567) since this region is least similar to the other gene family members. From the pool of selected 21 mer sequences, those with runs of more than 4 As or Ts were eliminated, and those with GC content between 30% and 50% were lengthened to 29 bp by adding the next eight bases in the genomic sequence. The TIGR *E. histolytica *Genome Project database [52] was used to check that each 29-bp sequence was unique to its gene, with non-unique ones eliminated. A minimum of four unique sequences were selected per gene. To create a scrambled control sequence, one of the selected sequences was chosen, and the bases were scrambled (each began with the AA dinucleotide); these sequences were then checked to confirm they matched nothing in the *E. histolytica *genome. In addition, a sequence targeted to the green fluorescent protein (GFP) was included as a control [30]. The chosen sequences, those ultimately transfected into *E. histolytica *HM1:IMSS trophozoites, are shown in Table 1. Constructs that did not successfully transfect are not shown.

### shRNA primer design

Primers were designed based on the method used by Gou et al (2003) [30] to yield PCR-generated shRNA constructs in a 2-step PCR process diagrammed in Figure 1. The final PCR product contained the *E. histolytica *U6 promoter followed by the sense strand of the hairpin, the 9 bp loop (TTCAAGAGA) [28], the antisense strand of the hairpin, and the U6 terminator sequence [30]. An *Apa*I restriction site (GGGCCC) was included between the 3' end of the U6 promoter and the beginning of the shRNA sequence [30]. To facilitate cloning of the PCR product into the expression vector, a *Hin*dIII site was added to the 5' end of the U6 promoter sequence, and a *Not*I site was added following the terminator sequence. The selected siRNA sequences, shown in Table 1, were used to design oligos to create shRNAs. Two rounds of PCR were employed to generate the final shRNA constructs, using one forward primer and two reverse primers, whose sequences are listed in Table 2. In the first round of PCR, the *E. histolytica *U6 promoter followed by the sense strand and the loop were generated using a forward primer amplifying the 5' end of the U6 promoter and a first reverse primer containing the sequence of the sense strand of the shRNA and the future loop (Figure 1A, Table 2). A second round of PCR created the completed shRNA construct: the product from the first round was used as a template, using the same forward primer as in the first round, and a second reverse primer containing the sequence of the loop, the antisense strand sequence, and the U6 termination sequence (Figure 1A, Table 2). The control GFP sequence [30] was used to design oligos for making a shRNA control construct. Sense strand sequences chosen to make the Igl, URE3-BP and EhC2A shRNA constructs successfully transfected into trophozoites are shown in Table 1, and PCR oligos used to amplify these sequences to generate shRNAs via PCR are shown in Table 2.

### PCR conditions for generating shRNAs

Initially, *E. histolytica *genomic DNA was used as a template for the first round of Igl shRNA PCRs. For the URE3-BP and EhC2A shRNA PCRs, the cloned U6 promoter was used as the PCR template: the Igl shRNA plasmids were digested with *Hin*dIII and *Apa*I and the U6 promoter was gel-purified using the QIAquick Gel Extraction Kit (Qiagen, Valencia, CA, USA). Two rounds of PCR were used to generate the shRNA constructs.

The first PCR round generated the sense strand of the hairpin and the loop. Reaction volumes of 40 μl were set up, each consisting of 0.6 μl SAHARA™ DNA polymerase (Bioline USA Inc., Taunton, MA, USA), 4 μl 10× SAHARA™ PCR buffer, 3.2 μl 50 mM MgCl_2_, 2 μl dNTP mix (stock 10 mM each), 0.4 μl U6 *Hin*dIII forward oligo (100 μM stock), 0.4 μl R1 oligo (100 μM stock), 1 μl (200 ng *E. histolytica *genomic DNA or 25 ng gel-purified digest of *Hin*dIII/*Apa*I U6 promoter), and 28.4 μl sterile water. Cycling conditions were as follows: 95°C for 8 minutes, 10 cycles of 95°C 45 sec, 40°C 1 min, 68°C 1 min 30 sec; 25 cycles of 95°C 45 seconds, 52°C 1 min, 68°C 1 min 30 sec, and a 5 min final extension at 68°C. 5 μl of each PCR product was subjected to agarose gel electrophoresis to check that the products were ~380 bp.

In the second PCR round, the first round PCR product was used as a template to add the antisense strand of the hairpin, the terminator sequence and the *Not*I site. Each 100 μl-volume reaction contained 2 μl SAHARA™ DNA Polymerase (Bioline USA Inc., Taunton, MA, USA), 10 μl 10× SAHARA™ PCR buffer, 8 μl 50 mM MgCl_2_, 5 μl dNTP mix (10 mM each), 0.8 μl U6 *Hin*dIII forward oligo (100 μM), 0.8 μl R2 oligo (100 μM), 2 μl PCR product from the first PCR round, and 71.4 μl sterile water. Cycling conditions were: 95°C for 8 minutes, 10 cycles of 95°C 45 sec, 18.5°C 1 min 30 sec, 68°C 1 min 30 sec; 30 cycles of 95°C 45 seconds, 55°C 1 min, 68°C 1 min 30 sec, and a 5 min final extension at 68°C. The low annealing temperature in the early cycles of the second PCR was used since the loop is the only overlap between the first round product and the second round reverse oligo. The second round PCR products were checked by agarose gel electrophoresis for products of the correct size (~420 bp). Sometimes a smaller product was present in addition to the correct size product in the final PCR product; this was ignored since it had no effect on the subsequent cloning steps. These final PCR products were ethanol-precipitated, then they and modified pGIR310 were digested with *Hin*dIII and *Not*I. The digested expression vector was gel-purified using the QIAquick Gel Extraction Kit (Qiagen, Valencia, CA, USA). The PCR products were ethanol-precipitated at -20°C overnight, resuspended, ligated into modified pGIR310, transformed into *E. coli*, and colonies screened. Plasmids with inserts were sequenced, and those with perfect U6 promoter and hairpin sequences were cultured, plasmids were isolated using the Qiagen HiSpeed Maxiprep Kit (Qiagen, Valencia, CA, USA), and transformed into HM1:IMSS strain trophozoites as described above.

### Western blotting

The Igl, URE3-BP, or EhC2A shRNA transfectants were grown in 25 cm^2 ^tissue culture flasks and selected beginning with 15 μg/ml of hygromycin, with the hygromycin level increased every 24 hours until the final level of selection was reached, and this level was maintained for 48 hours before harvesting. The GFP control, all three Igl, and the URE3-BP (350–378) transfectants were selected with 100 μg/ml, the URE3-BP (580–608) shRNA transfectants with 75 μg/ml, and the EhC2A samples with 90 μg/ml hygromycin. The final concentration of hygromycin selection differs since the selection was increased until the desired level of knockdown was achieved. There were three biological replicates per shRNA transfectant, and one for the HM1:IMSS nontransfected trophozoites. Trophozoites were harvested as described above for transfection, counted, resuspended in ice cold Lysis Buffer (150 mM NaCl, 50 mM Tris, 5× Sigma protease inhibitor cocktail (P2714) (Sigma-Aldrich, St. Louis, MO, USA), 25 μg/ml E-64 (Sigma-Aldrich, St. Louis, MO, USA)) at an initial concentration of 2 × 10^6^–5 × 10^6 ^amebae/ml, and lysed by sonication by pulsing twice for 10 seconds each with a 10 second rest on ice between pulses. Protein was quantified and sample lysates were diluted to the same protein concentration, were serially-diluted 1:2, 1:4, and 1:8 with Lysis Buffer, and were subjected to SDS-PAGE on 12% (Igl) or 15% (URE-BP and EhC2A) gels. All sample lysates and dilutions were done in triplicate (technical replicates). Gels were transferred to PVDF membrane, membranes were cut in half so each half could be probed separately, were blocked in 5% milk, and incubated with either antibodies against Igl1, URE3-BP, EhC2A, or control antibodies against actin (anti-actin from Santa Cruz Biotechnology (Santa Cruz Biotechnology, Santa Cruz, CA) or Sigma (Sigma-Aldrich, St. Louis, MO, USA)).

The ECL kit from Roche (Roche Applied Science, Indianapolis, IN, USA) was used to treat membranes after secondary antibody incubation, bands were visualized on film, film images were electronically scanned, and Scion Image Beta 4.0.3 software (Scion Corporation, Frederick, MD, USA) was used to quantify band intensity. The background value was subtracted from each band value, the ratio of Igl, EhC2A, or URE3-BP band value to the control actin band value was taken for normalization, and then the sample shRNA transfectant lines were compared to the control GFP shRNA or scrambled shRNA transfectant line. HM1:IMSS nontransfected samples were also included. Values for each shRNA transfectant were averaged, and the SE for each average was calculated using the total number of biological replicates multiplied by the number of technical replicates. Statistical analysis was performed using Student's t test (two-tailed) or ANOVA. The GraphPad QuickCalcs P-value calculator was used to calculate the P-values [53].

### Isolation of total RNA

Igl, URE3-BP, and control GFP transfectant shRNA lines were selected with hygromycin as described above for Western blotting, and samples were collected and frozen in TRIzol reagent (Invitrogen, Carlsbad, CA, USA) at -80°C for RNA isolation at the same time as those harvested for crude lysate for protein analysis. Total RNA isolated from each shRNA transfectant and nontransfected HM1:IMSS sample using TRIzol reagent (Invitrogen, Carlsbad, CA, USA) was treated with RNase-free recombinant DNase I (Roche, Indianapolis, IN, USA) for 30 minutes at 37°C, and purified on RNeasy columns using the RNeasy Mini kit as per the manufacturer's instructions (Qiagen, Valencia, CA, USA). Five μg RNA per sample was reverse-transcribed using SuperScriptII (Invitrogen, Carlsbad, CA, USA) and anchored oligo dT, including samples with no reverse transcriptase added (no-RT controls). To check samples for residual DNA contamination in the no-RT controls, each was screened with primers specific for the Jacob cyst-specific gene [35]. If residual DNA contamination was observed, the RNA was treated again with DNase I as above, re-purified on RNeasy columns, and re-screened.

### Quantitative reverse-transcription real-time PCR (qRT-PCR)

After the screen for residual DNA contamination was completed, the cDNA was quantified, and sample cDNAs were diluted to 100 ng/μl. HM1:IMSS cDNA was also serially-diluted for making a standard curve. All primers used for qRT-PCR in this study were selected to amplify <400 bp sections of mRNA. Amplification of actin [35] was performed for use as a normalization control. Oligo sequences used in qRT-PCR are shown in Table 3. Each oligo pair was checked using the *E. histolytica *genomic database [52] to validate that only the gene intended would be amplified, except for actin and Jacob, which were designed to detect all family members [35]. An MJ Research Opticon2 DNA Engine (Bio-Rad, Hercules, CA, USA) was utilized for all qRT-PCR runs. ~200 ng of each sample or control cDNA, or serially-diluted HM1:IMSS cDNA for standard curves, was added to each sample well in a 96-well plate for each set of amplifications. cDNA from each biological replicate was run in quadruplicate (technical replicates), and there were three biological replicates per transfectant line, except for HM1:IMSS nontransfected samples, which had one biological replicate. No-RT controls were also included for each set of samples. Each well contained in addition to the cDNA: 1.25 U HotStarTaq (Qiagen, Valencia, CA, USA), 1× HotStarTaq PCR Buffer, 0.5 mM MgCl_2_, 200 μM each dNTP, 1 μM each oligo, 1:10,000 dilution of Sybrgreen (Invitrogen, Carlsbad, CA, USA), 0.15% Triton X-100, and water to a volume to 25 μl per well.

qRT-PCR cycling conditions were 95°C for 15 minutes, followed by 40 cycles of 95°C 30 s; 54°C 30 s; 72°C 45 s, followed by one cycle of 72°C for 3 min. At the end of amplification, a melt curve was performed from 70°C to 95°C, increasing 0.2°C every cycle with a 5-second hold. The C_T _values were averaged for each oligo pair for each set of technical replicates, and sample values were normalized to the housekeeping gene actin. The GFP shRNA transfectant line was used as a baseline control for comparison to the URE3-BP and Igl shRNA transfectant lines; HM1:IMSS samples were included as a secondary control. The differences in gene expression for the URE3-BP and Igl transfectant lines as compared to the GFP transfectant line were calculated by using both the relative standard curve and the comparative C(t) method (ΔΔ C(t) method) [54,55]. Statistical analysis was performed using Student's t test (two-tailed), groups were also compared using ANOVA, and the GraphPad QuickCalcs P-value calculator [53] was used to calculate P-values.

### Isolation of small RNAs

Three of the Igl shRNA transfectant lines, Igl (1198–1226), Igl (2412–2440), and Igl (2777–2805), as well as the two PATMK knockdown shRNA lines, PATMK (2273–2301) and PATMK (3552–3580), and the PATMK scrambled control [39], were grown in 25 cm^2 ^tissue culture flasks, and selected with 30 μg/ml hygromycin, since this level of selection had yielded substantial knockdown of PATMK [39]. Small RNAs were isolated from each sample as well as control nontransfected HM1:IMSS trophozoites using Ambion's mirVana™ miRNA Isolation Kit (Applied Biosystems/Ambion, Austin, TX, USA) as per the manufacturer's instructions.

### Northern blotting of small RNAs

Oligo probes were designed to match the sense or antisense strands of each hairpin. Fifty μg of small RNAs were loaded per lane on a 12% denaturing acrylamide gel and transferred to Hybond™-N+ nylon membrane (Amersham Biosciences/GE Healthcare Biosciences Corp, Piscataway, NJ, USA) as per the manufacturer's instructions. rRNA bands were analyzed to insure equal RNA loading. Oligo probes matching to the sense or antisense strands of the hairpins were end-labelled with ^32^P and were hybridized with each corresponding sample blot strip overnight at 37°C overnight, washed with low and medium stringency conditions, and exposed overnight to film.

## Authors' contributions

ASL designed and performed the majority of the experimental work, including the design of shRNA oligos, cloning of shRNA vector constructs, transfection and expression analyses in *E. histolytica*, and wrote the manuscript. HM conducted all experiments with EhC2A and helped edit the manuscript. KRG helped design and clone the shRNA vectors for URE3-BP and analyze the resulting transfectants. HZ and US conducted the small RNA analysis. WAP conceived of this study and oversaw its coordination, design and analysis.
